# A Study on the Protective Effect of sRAGE-MSCs in a Rodent Reperfusion Model of Myocardial Infarction

**DOI:** 10.3390/ijms232415630

**Published:** 2022-12-09

**Authors:** Delger Bayarsaikhan, Govigerel Bayarsaikhan, Jaewon Lee, Bonghee Lee

**Affiliations:** Lee Gil Ya Cancer and Diabetes Institute, School of Medicine, Gachon University, Incheon 21565, Republic of Korea

**Keywords:** acute myocardial ischemia, macrophage, AGE-albumin, cardiomyocyte death, soluble RAGE, MSCs, gene editing

## Abstract

Acute myocardial infarction (AMI) is one of the major leading causes of death in humans globally. Recently, increased levels of recruited macrophages and AGE-albumin were observed in the hearts of humans and animals with acute myocardial infarction. Thus, the purposes of this study were to investigate whether the elevated levels of AGE-albumin from activated macrophage cells are implicated in ischemia-induced cardiomyocyte death and to develop therapeutic strategies for AMI based on its underlying molecular mechanisms with respect to AGEs. The present study demonstrated that activated macrophages and AGE-albumin were observed in heart tissues obtained from humans and rats with AMI incidences. In the cellular model of AMI, it was found that increased expression of AGE-albumin was shown to be co-localized with macrophages, and the presence of AGE-albumin led to increased expression of RAGE through the mitogen-activated protein kinase pathway. After revealing cardiomyocyte apoptosis induced by toxicity of the AGE-RAGE system, sRAGE-secreting MSCs were generated using the CRISPR/Cas9 platform to investigate the therapeutic effects of sRAGE-MSCs in an AMI rat model. Gene-edited sRAGE-MSCs showed greater therapeutic effects against AMI pathogenesis in rat models compared to mock MSCs, and promising results of the functional improvement of stem cells could result in significant improvements in the clinical management of cardiovascular diseases.

## 1. Introduction

Rapidly increased progress in stem cell science and advances in gene engineering are bringing together a wide range of evolutions in biomedical sciences. Consistent study results have shown tremendously positive effects against numerous intractable diseases, and many of them are promoted to the clinical stage in human cases [1]. Among these intractable diseases, cardiovascular disorders, especially myocardial infraction, has a relatively high mortality rate compared to others. According to global estimates, 13 million people died from cardiovascular diseases (CVD) in 2010, with ischemic heart disease and stroke accounting for the majority of deaths [2]. The most effective and necessary therapy for acute myocardial infarction (AMI) is reducing ischemic injury and limiting the size of myocardial infarction using either thrombolytic therapy or primary percutaneous coronary intervention. However, the process of restoring blood flow can itself induce cardiomyocyte death, which is known as myocardial reperfusion injury [3,4,5]. In addition to traditional postconditioning approaches, stem-cell-based methods are also considered extensively for ischemic and reperfusion-induced damages. As an example, mesenchymal stem cells (MSCs), bone-marrow-derived mononuclear cells, peripheral blood stem cells, endothelial progenitor cells, and neuronal stem cells have been examined for their therapeutic potential against stroke-induced damage, due to their capacity to release endogenous substances and paracrine factors as cardiomyocyte repair and protection agents [6,7,8]. Although some systemic analyses found stem cell transplantations to be limited or modest in terms of their effectiveness and ethical issues, improvements are still necessary for their use in clinical practice [9,10].

After myocardial reperfusion injury, damaged cardiomyocytes secrete danger signals such as HIF1α, MCP-1, and IL1-β, and then those cytokines induce activation and accumulation of macrophages in the infarcted area. In fact, this region of ischemic reperfusion heart injury also displays a highly elevated appearance of activated macrophage cells, which leads to the induction of further inflammation and left ventricular remodeling by secreting cytokines and proteins [11,12,13,14,15,16]. Macrophages, Kupffer cells, and microglial cells are derived from hematopoietic monocytes, and recent studies have demonstrated that activated microglial cells can secrete/synthesize advanced glycation end products (AGEs) with the presence of their receptor (RAGE) and promote cell death and inflammatory reactions [17,18].

AGEs play a central role in biomarkers for a variety of diseases, including neurodegenerative disorders, diabetes and diabetes-induced organ failures, alcoholic liver and brain damages, and others [19,20,21]. In addition, AGEs were observed in the hearts of humans and animals with acute myocardial infarction, and the increased activation of RAGE by AGEs showed increases in cell apoptosis through different biochemical pathways such as oxidative stress and hyper responsiveness to the macrophages during inflammation [22,23,24,25]. RAGE is a member of the immunoglobulins, and AGE–RAGE bindings stimulate the activation of diverse signaling cascades—for example, mitogen-activated protein kinases and phosphoinositol-3 kinase/Akt to Jak/Stat pathways—and finally lead to the apoptotic pathway [26,27,28,29].

The present study aimed to study whether the AGEs from activated macrophage cells are a key inducer of cardiomyocyte death in human and rat AMI-IR.

## 2. Results

### 2.1. AGE-Albumin Synthesis and Secretion in AMI Hearts of Humans and Rats

**Figure 1:**
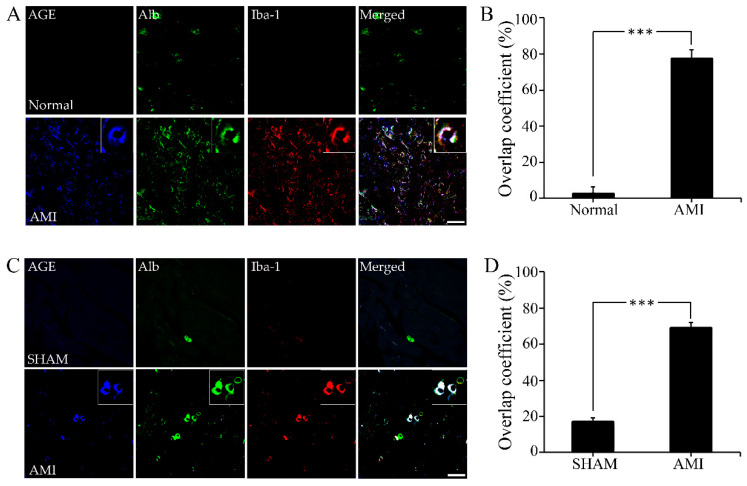
Co-localization of AGE-albumin and activated macrophage cells in the ischemic reperfusion injured heart of human and rat. (**A**) Triple-labeled immunostaining of AGE (blue), albumin (green), and Iba1 (red, activated microglial cell marker) in the left ventricle of human (**A**) and rat (**C**). Merged image shows that AGE (blue), albumin (green), and Iba1 (red, activated microglial cell marker) were co-localized mostly in infarcted area of heart. Fluorescence expression level (**B**,**D**) and co-localization coefficient analyzed by densitometry analysis software using Zen software (Zeiss). Scale bar = 50 μm; ***, *p* < 0.001.

Localized distribution of activated macrophages and AGE-albumin in the ischemic reperfusion injured tissues of human and rat hearts were studied to investigate their correlation in the pathogenesis of AMI using triple-labeled immunostaining. Interestingly, most of the AGEs were detected within activated macrophage cells and co-localized with albumin. This result demonstrates that AGE-albumin could be a major AGE product in activated macrophage cells in the hearts of AMI patients (Figure 1A). Furthermore, AGE-albumin levels were dramatically higher in the heart tissue samples of AMI subjects (n = 3) than in healthy subjects (N = 3), as shown in Figure 1B. Similar experiments were performed on heart tissues of rats collected from healthy subjects and post 28 days of AMI, and the results showed that most of AGE-albumin co-localized with activated macrophages in damaged hearts following ischemic reperfusion (Figure 1C,D).

**Figure 2:**
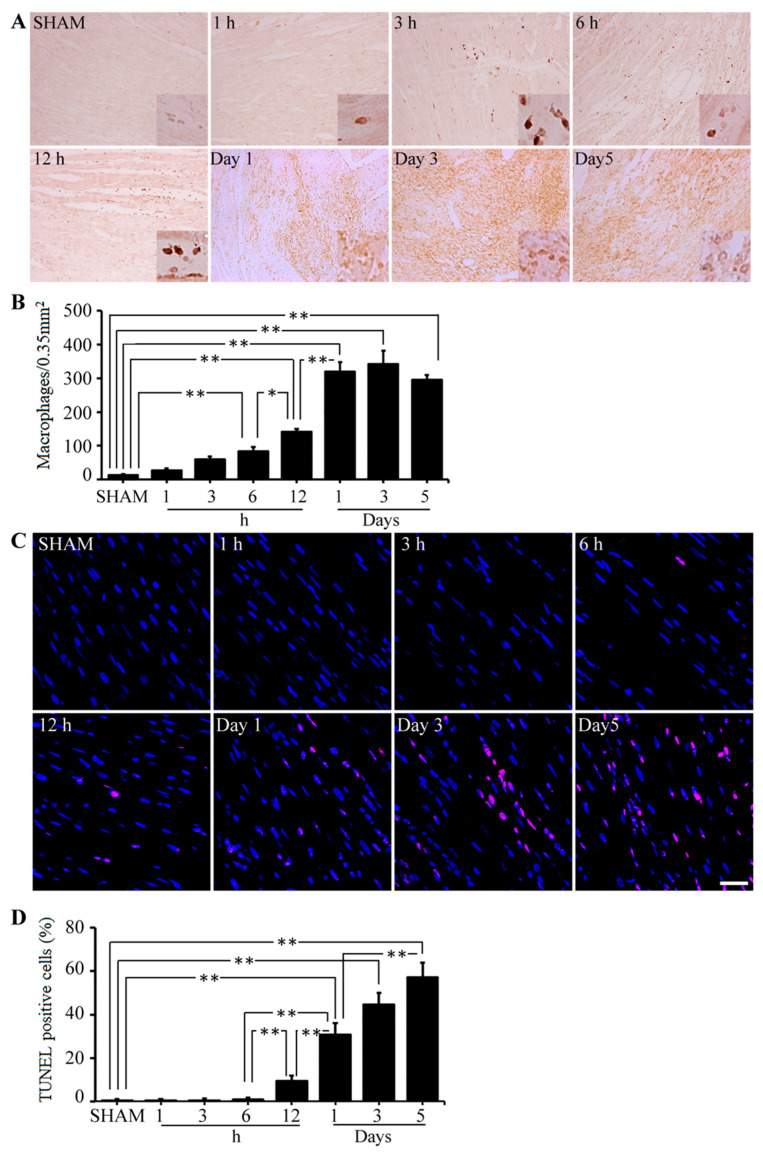
Time-dependent distribution of activated macrophages and apoptotic cardiomyocytes in the left ventricle of the sham and ischemic reperfusion injured heart of rats. (**A**) Iba-1-positive macrophage cells were measured by immunohistochemical staining in control or ischemic reperfusion injured rat hearts at different time periods. (**B**) A number of Iba-1-positive cells in the heart of control and ischemic reperfusion injured rats. (**C**) TUNEL staining used to detect apoptotic cardiomyocytes in control or ischemic reperfusion injured rat hearts at different time periods. (**D**) A percent of TUNEL-positive cells in the heart of control and ischemic reperfusion injured rats. Scale bar = 50 µm. In the statistical analysis, a one-way ANOVA was performed, and Tukey’s test was used to compare means: *, *p* < 0.05; **, *p* < 0.01.

In advance, LAD coronary artery ligations on rats were performed to determine whether AMI-induced cardiomyocytes were correlated with macrophage activation. According to the study, there were no significant differences in the numbers of activated macrophages in heart tissues of sham and AMI-induced rats within the first hour of surgery; however, the number increased dramatically within 3 h to several days of surgery in the AMI-induced rats’ hearts. Based on immunostaining results and the number of detected activated macrophages per 0.35 mm^2^ area of the ischemic heart area in rats, the highest levels of activated macrophages were observed on day 3 post surgery, and then they were decreased over the following days (Figure 2A,B). The TUNEL analysis was conducted in conjunction with immunostaining for monitoring the apoptosis rate of cardiomyocytes in this study. With respect to the time intervals after ischemic reperfusion procedures, a statistically significant positive correlation (*p* < 0.05) was found between the number of activated macrophages and apoptotic cardiomyocytes in rats, as shown in Figure 2C,D.

**Figure 3:**
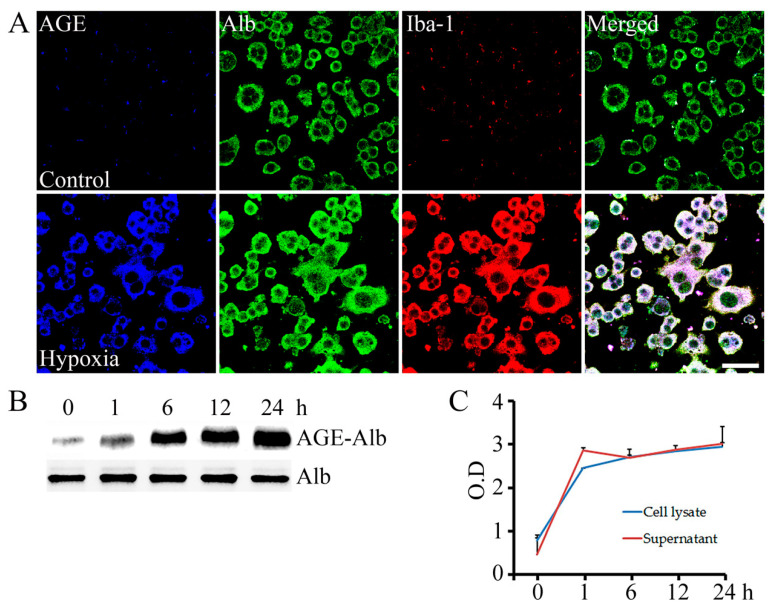
Increased synthesis and secretion of AGE-albumin in activated macrophage cells. (**A**) Triple-labeled confocal microscopic image analyses were used to study the distribution and relative levels of AGE (blue), albumin (green), and Iba-1 (red) in rat macrophage cells after treated with hypoxia-exposed cardiomyocyte conditioned medium. Scale bar = 50 μm. (**B**) The dose-dependent increases in AGE-albumin in total lysates of RAW cells treated with 0, 1, 6, 12, or 24 h hypoxia-exposed cardiomyocyte conditioned medium for 48 h were determined by co-immunoprecipitation. (**C**) A graph illustrating the dose-dependent changes in intracellular (cell lysates) and extracellular (culture supernatant) levels of AGE-albumin in RAW cells treated with 0, 1, 6, 12, or 24 h hypoxia-exposed cardiomyocyte conditioned medium for 48 h, as determined by ELISA.

As shown in Figure 1, activated macrophages and AGE-albumin are co-localized in ischemic heart tissues. Accordingly, a further objective of this study was to determine whether activated macrophages synthesize and release AGE-albumin, aimed at understanding the mechanism underlying this coexistence between activated macrophages and AGEs during pathogenesis of AMI using hypoxia-induced cardiomyocytes (H9C2 cell line) to mimic the ischemic process at the cellular level. Consequently, 1 × 10^6^ number of cardiomyocytes (H9C2 cell line) were exposed to hypoxia damage for 1, 6, 12, and 24 h, and corresponding media were collected and transferred to the rat macrophage cells (RAW 267.4) to activate them by hypoxia-associated cytokines and biomolecules [17]. The level of the AGE-albumin was controlled by ICC, Western blot, and ELISA analysis, and the result was that AGE-albumin levels were dramatically increased due to rat macrophage cell activation (Iba-1 positive) by hypoxic damage in vitro. The co-localizations of AGE-albumin and activated macrophages in Figure 1 and Figure 3A indicate that macrophages are capable to synthesize and secrete AGEs under ischemic reperfusion injury. Furthermore, co-immunoprecipitation and ELISA measurement on the cell supernatant and total lysate showed that rat macrophage cells contributed to the synthesis and increased level of AGEs under the AMI mimicked environment. In the cellular model, intracellular (cell lysates) and extracellular (culture supernatant) levels of AGE-albumin in RAW cells treated with 1 to 24 h of hypoxia-exposed cardiomyocyte conditioned medium for 48 h were increased up to 2.5–3 times (Figure 3B,C). Moreover, ANOVA followed by Tukey’s post hoc test indicated a significant difference between the 6th hour and 24th hour results of the cell lysate sample. As opposed to these results, the results of the supernatant samples at six, twelve, and twenty-four hours were not significantly different. In the supernatant, the AGE level stabilized after 6 h, while in the cell lysate, it increased significantly for the first hour before slowly increasing from the sixth hour (Figure 3C).

### 2.2. Post-Ischemic Reperfusion Injury Increases RAGE in AGE-Dependent Manner

**Figure 4:**
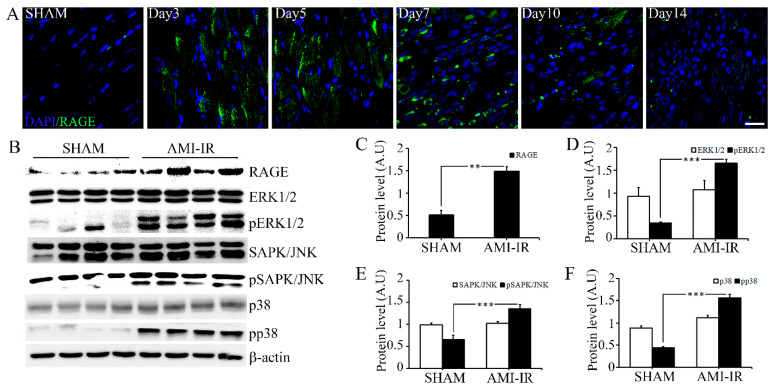
The relative level of RAGE and MAPKs in ischemic reperfusion injured rat heart. (**A**) RAGE expression is shown in double-labeled confocal images: RAGE (green) and DAPI (blue) in the left ventricle of sham and ischemic reperfusion injured hearts of rats at day 3, 5, 7, 10, and 14 in the border area. (**B**) Immunoblot analysis was performed to determine the expressed levels of RAGE, ERK1/2, p38, SAPK/JNK, pERK1/2, pp38, pSAPK/JNK, and β-actin, used as internal controls for equal protein loading of each lane. (**C**–**F**) Densitometry analyses of MAPK proteins were evaluated using the Image-J software. Scale bar = 50 µm; **, *p* < 0.01; ***, *p* < 0.001.

There is growing evidence that the AGE-RAGE axis plays a major role in the pathogenesis of ischemia reperfusion injury, and this study shows that AGE-albumin induces significant changes in the expression of RAGE in rats with AMI compared to controls (Figure 4A). We examined changes in the relative levels of RAGE and mitogen-activated protein kinases (MAPKs) in heart tissues collected from rats induced with AMI and sham, since stress-activated MAPKs and increased mitochondrial calcium influx are critical for initiating apoptosis [30,31]. In rats with ischemic reperfusion injury, immunoblot analysis revealed significant increases in RAGE and phosphorylated extracellular regulated kinases, including pSAPK/JNK, pERK1/2, and pp38 (Figure 4B–F). Overall, the results show that post-ischemic reperfusion injury promotes the secretion of AGE-albumin through macrophage activation, and furthermore, increases in AGE-albumin lead to increased expression of RAGE, which promotes cell apoptosis through AGE-RAGE signaling pathways.

**Figure 5:**
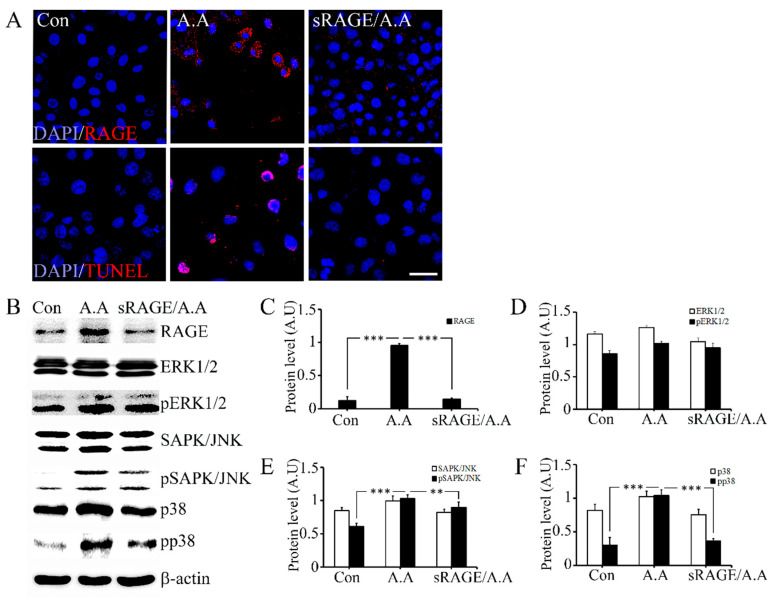
Protective effect of sRAGE on AGE-albumin-induced cardiomyocyte death by decreasing RAGE level. (**A**) RAGE expression is shown in double-labeled confocal images: RAGE (red) and DAPI (blue) using H9C2 cell before and after exposing AGE-albumin or co-treated with AGE-albumin and sRAGE. Cardiomyocyte cell death was evaluated by double staining with TUNEL (red) and DAPI (blue). (**B**) Immunoblot analysis of cardiomyocytes lysates after AGE-albumin or AGE-albumin with sRAGE co-treatment. RAGE expression was increased after AGE-albumin treatment but decreased after co-treatment with sRAGE. In MAPK analysis, pp38 and pSAPK/JNK were increased after AGE-albumin treatment but decreased after co-treatment. (**C**–**F**) Densitometry analyses of MAPK proteins were evaluated using the Image-J software. Scale bar = 50 µm; **, *p* < 0.01; ***, *p* < 0.001.

The immunostaining results of AGE-albumin-treated rat cardiomyocytes with or without the co-treatment of sRAGE demonstrated that the level of RAGE expression in cardiomyocytes treated with AGEs was higher than in those exposed to co-treatment of AGEs and sRAGE (Figure 5A). It was found that apoptosis in cells dependent on AGEs can be drastically reduced by sRAGE, which is regarded as a competitive inhibitor of RAGE (Figure 5B). A comparison of cardiomyocytes treated with and without AGEs revealed that phosphorylated and non-phosphorylated MAPK proteins were more likely to be expressed in AGEs alone but were decreased when combined with AGEs and sRAGE (Figure 5C–F).

### 2.3. The sRAGE-Secreting MSC Is a Novel Therapy for AMI

**Figure 6:**
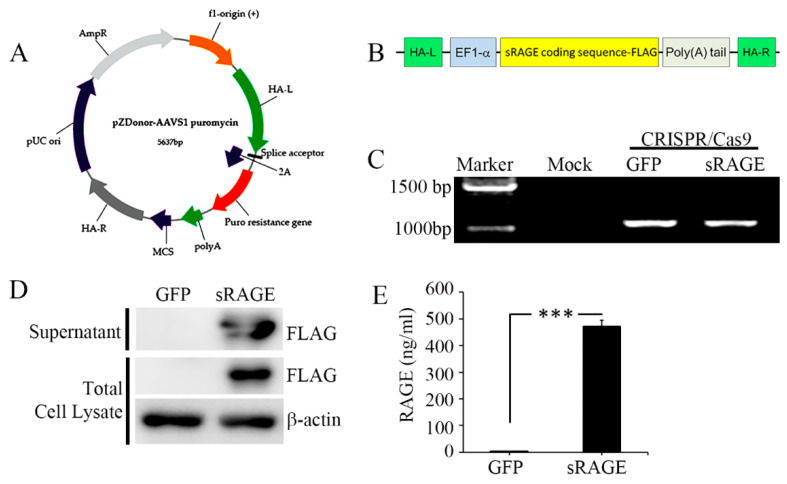
sRAGE-secreting MSC line characterization. (**A**) The illustration picture represents the gene information of pZDonor-AAVS1 puromycin vector. Each arrow describes a certain gene. (**B**) The illustration of sRAGE insertion coding sequence. (**C**) Genome integration was confirmed with genomic DNAs of MSCs which were transfected with mock, GFP, and sRAGE-containing pZDonor-AAVS1 plasmids. (**D**) Western blot analysis of supernatant and extract from MSCs transfected with GFP (lane 1) and FLAG-tagged sRAGE in pZDonor-AAVS1 vector (lane 2). β-actin loaded as a positive control. (**E**) The secretion of human sRAGE levels was confirmed with ELISA after puromycin selection; and statistical analysis was performed using Student’s *t*-test; ***, *p* < 0.001.

In our next step, we developed sRAGE-secreting MSCs as a novel therapy for acute-myocardial-infarction-induced ischemic reperfusion injury (AMI-IR), and the effectiveness of the gene-edited cell line was confirmed on the AMI-IR rat models. The cell line was obtained from umbilical cord blood driven using the CRISPR/Cas9 (Figure 6A,B) technique in the NEON transfection system, and efficiency of the gene editing was confirmed by junction PCR (Figure 6C). Additionally, sRAGE secretion and synthesis of the gene-edited cell line was measured by Western blotting with a selective antibody (Flag), in which the Flag is expressed by only sRAGE-MSC with pzDonor transfection (Figure 6D). However, almost no sRAGE secretion was found for wild-type mock-MSCs (0.3 ng/mL), but after the gene modification, the sRAGE secretion level was tremendously increased up to 471.6 ng/mL in the cell culture medium (Figure 6E).

**Figure 7:**
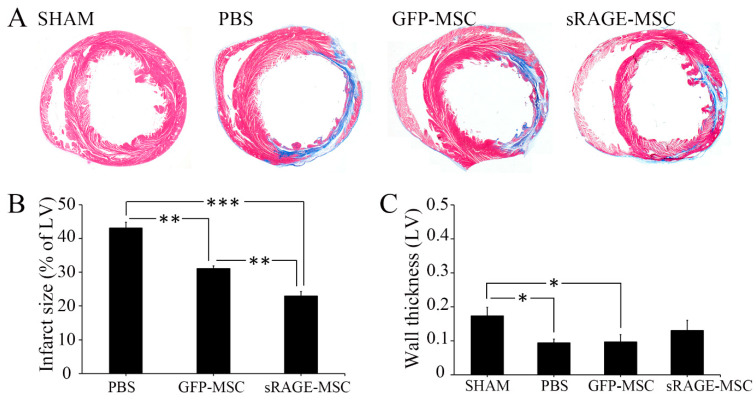
Soluble RAGE-secreting MSC protection of ischemia-mediated cardiomyocyte death by decreasing RAGE level. (**A**) Representative Masson’s trichrome staining revealed left ventricular fibrosis 4 weeks after AMI (magnification: 10X). Red color indicates viable myocardium; blue color indicates fibrosis due to infarction damage. (**B**) Infarct size was expressed as a percentage of ischemic area (**C**) and as the length of wall thickness; *, *p* < 0.05; **, *p* < 0.01; ***, *p* < 0.001.

After validating the integration of sRAGEs in MSCs, their therapeutic effect was examined on AMI-developed rat models as described in the Section 4. Fibrosis of the heart is one of the most well-known pathological processes implicated in post-AMI reactions [32]. AMI-induced fibrosis in cardiac tissues was investigated in this study by Masson’s trichrome staining on rat cardiac tissues obtained 28 days after the AMI-inducing procedure to detect the protective effect of sRAGE, which is secreted from MSCs. In the present study, the results demonstrate that the size of the infarcted area in heart tissue was reduced in the sRAGE-MSC-treated group compared to the PBS- and GFP-MSC-treated groups (Figure 7A). Image J software was also used to calculate the percentage of fibrosis and wall thickness in the LV cross-sectional area (Figure 7B,C). The results showed that the percentage of the cardiac infarct size of the LV cross-section was highest for AMI-inducing procedures with PBS and lowest for AMI-inducing surgery in conjunction with transplantation of sRAGE-MSCs. Analysis of LV wall thickness after AMI-inducing procedures revealed that the thickness was highest for sham and lowest for PBS injection. However, both wild-type and sRAGE-secreting MSCs protected against AMI-induced decreases in cardiac wall thickness, but functionally improved MSCs showed greater activity. In other words, the therapeutic and protective capacities of sRAGE-secreting MSCs were much higher than wild-type MSCs against cardiac fibrosis. This implies that the functional improvement of the stem cells could bring promising beneficial effects in the clinical management of AMI patients.

## 3. Discussion

Our previous studies reported that microglial cells secret AGE-albumin during several neurodegenerative disorders, including Alzheimer’s disease, Parkinson’s disease, and alcoholic dementia brain damage, and explained how the immune cells stimulate the disease progression under the hyperactivated condition [33,34,35]. Through this study, we extended our previous knowledge with two main pathways. The results presented in this study contribute to understanding how macrophage activation is involved in AMI-induced cardiomyocyte apoptosis and illustrate the therapeutic effectiveness of sRAGE-secreting stem cells for curing and reducing the severity of AMI incidences.

Firstly, we predicted the mechanism for AMI-induced cell apoptosis via AGE and RAGE interaction in the heart using human and rat tissues. According to our hypothesis, this process consists of three steps: (i) macrophage activation through AMI signaling, (ii) AGE-albumin secretion by activated macrophages, and (iii) RAGE-AGE stress-induced cardiomyocyte death. It has been interpreted that in healthy humans, macrophage levels are relatively low, but that after myocardial infarction, macrophage subtypes and numbers increase, which leads to fibrosis through chemokine-dependent pathways [36]. Moreover, many studies on AMI demonstrated that myocardial cells release certain cytokines and cytotoxic agents under ischemic conditions, which recruit macrophages and induce its constative activation [11,12,13,14,15,16]. The interesting question is whether AGEs are secreted through resident or recruited macrophages. The recent review summarized that non-resident macrophages replace resident macrophages in the heart and promote cardiac fibrosis in response to inflammation [37] Along with these findings, our study demonstrates that macrophage cells are recruited to cardiac damaged areas and secrete/synthesize AGE-albumin in both human and rat subjects with AMI incidences. As one of the most binding cellular receptors, RAGE is highly selective for AGEs, and AGE-RAGE-induced stress was linked to the pathogenesis of a wide range of diseases by stimulating the uptake of certain toxicants into macrophages, which may increase the risk of cardiovascular disorders [38]. To decrease the toxicity of the AGE-RAGE system several approaches were developed, including the application of gene engineering technologies to knock out the cellular RAGE receptor and the utilization of anti-AGE/RAGE strategies, including those with an AGE cross-link breaker (i.e., alagebrium), those with an AGE production blocker (i.e, aminoguanidine), those blocking AGE-RAGE signaling with antibodies and antioxidants, or those introducing RAGE antagonists [39,40,41]. The soluble form of RAGE, known as sRAGE, acts as a natural antagonist of RAGE by decoying RAGE ligands and inhibiting RAGE-dependent cellular responses [42]. As demonstrated in other studies, sRAGE levels are dynamically changing with the time intervals of AMI. In STEMI patients, it was found that sRAGE levels were high in the first day of AMI and, starting from the following day, reduced significantly, and the reduced levels were associated with long-term cardiac dysfunction and infarct size [43]. Decreases in plasma sRAGE were demonstrated by another research group as well [44]. The therapeutic effects of sRAGE have been demonstrated for various disorders related to AGE-RAGE, including inflammatory disorders, atherosclerosis, myocardial ischemia reperfusion, neutrophilic asthma, atrial fibrillation, and others [45,46,47,48]. There are different strategies that can indirectly lower AGEs, despite the application of sRAGE. For instance, a statin therapy may inhibit AGE-RAGE-mediated pathogenesis by lowering cholesterol levels and increasing levels of sRAGE, as suggested by in-vitro experiments [49]. However, Falcone and colleagues surveyed 330 patients with acute coronary syndromes, including MI and unstable angina, and found that sRAGE plasma levels were significantly lower in these patients, and that statin therapy did not affect sRAGE level [50].

In conjunction with recent advances in genome engineering technologies, stem-cell-based strategies have been introduced into the clinical management of cardiovascular disorders as a safe and efficient therapeutic approach. For example, MSCs integrated with sRAGE via the CRISPR/Cas9 system have been shown to protect neurons from apoptosis during neurodegenerative disorders and enhance immunoregulatory functions during autoimmune arthritis [51,52]. In this study, we introduced sRAGE-secreting MSCs as a potential therapeutic approach against myocardial infarction using the CRISPR/Cas9 platform and a rodent model. It was found that functionally improved sRAGE-MSCs show higher protection from cardiac wall thickening and fibrosis during the pathogenesis of myocardial infarction than wild-type MSCs. The advantages of integrating sRAGE into MSCs could be explained by several factors. As a first point, MSCs are known for their safety and effectiveness in cardiac regeneration [53]. They are capable of interacting with the immune system to decrease inflammatory reactions and to promote the healing process and capable of differentiating into cardiomyocytes, vascular smooth muscle cells, or vascular endothelial cells. In addition, human clinical studies have shown that MSC transplantation after AMI significantly improves left ventricular systolic function [54,55,56]. Secondly, sRAGE shows an inhibiting role for the toxicity of the AGE-RAGE system and possesses a very short half-life in in vivo, approximately 2,98 h, while pathogenesis followed by AMI takes a longer time [57].

## 4. Materials and Methods

### 4.1. Human Heart Tissue

Heart tissues from normal and acute myocardial infarction subjects were obtained from the Korean National Forensic Centre. Tissues were collected from the left ventricle and directly fixed in 10% formalin buffer overnight and transferred to dehydration procedure. Dehydration steps consist of 70% ethanol—overnight at 4 °C, 80% ethanol—1 h at room temperature, 90% ethanol—1 h at room temperature, and 100% ethanol—2 times each for 1 h at room temperature. After dehydration, tissues were cleared with xylene—2 times each for 1.5 h and embedded in paraffin at 60 °C. The heart tissue collection and usage were approved by the Ethics Committee of the Gil Hospital, Incheon, and Korea (LCDI-2017-0093).

### 4.2. Cell Culture

Rat cardio myoblast cells (H9C2) and rat leukemic monocyte macrophage cells (RAW264.7) were used for the in vitro studies. RL4, H9C2, and RAW264.7 cells were grown in Dulbecco’s modified Eagle’s medium (Hyclone, SH30243.01; Logan, Utah, USA) containing high glucose concentration and supplemented with 10% fetal bovine serum (Gibco, 16000-044, New York, NY, USA) and 1% penicillin-streptomycin (Thermo Fisher, 15070063; New York, NY, USA) at 37 °C under 5% CO_2_.

To treat stem cells in the AMI-IR animal model, umbilical cord blood MSCs (UCB-MSC; obtained from Medi-post) were chosen. UCB-MSCs were grown in α-MEM medium (Gibco) supplemented with 10% fetal bovine serum (Gibco, 16000-044; New York, NY, USA) and 1% penicillin-streptomycin (ThermoFisher, 15070063; New York, NY, USA) at 37 °C under 5% CO_2_. 

To analyze the AGE-dependent activity of RAGE, we treated the H9C2 rat cardiomyocytes with 800 ug/mL AGE-albumin (Sigma, A8301; Saint Louis, MO, USA) with and without the co-treatment of 400 ng/mL soluble RAGE (sRAGE; obtained from Sigma, BioVender R&D, RD172116100; Mokrá Hora, Czech Republic) and monitored the RAGE expression level.

### 4.3. Generation and Characterization of sRAGE-Secreting MSC

To generate sRAGE-secreting UCB-MSCs, transfection was conducted with CRISPR/Cas9 and gRNA, which target the safe harbor site of adeno-associated virus integration site 1 (AAVS1). The nucleofection was performed once in 10 µL each in conditions of a voltage of 1000 pulse width 30 two times. Cells were seeded in 6-well plates, and each contained 8 × 10^5^ cells. The transfected cells were incubated at 37 °C for 4 days for cell maintenance, followed by 5 days of 1 µg/mL puromycin treatment to select cells with genome integration. Media were changed every two days and maintained for further experiments.

### 4.4. Myocardial Infarction Modeling and sRAGE-MSC Transplantation

Male Sprague-Dawley rats weighing 290–330 g (8–9 weeks of age) were induced in the MI/R procedure as previously described [58]. Briefly, rats were intubated and ventilated with a volume-cycled small-animal ventilator. Anesthesia was maintained during the operation with 5% isoflurane. The left anterior descending coronary artery (LAD) was identified and then the vessel was ligated by 6-0 polypropylene for 40 min. After reperfusion, three separate intramyocardial injections of 10 µL of phosphate buffered saline (PBS) with or without GFP-MSC and sRAGE-MSC cells (1 × 10^6^) were delivered into the peri-infarct and infarcted zone using Hamilton syringe. The muscle layer and skin were closed and allowed to recover. A sham-operated group of animals underwent the same experimental procedure but without ligation and cell transplantation. To prevent graft rejection, rats receiving cell transplantation were administrated with cyclosporine A (10 mg/kg/day). All animal experiments were approved by the Institute Animal Care and Use Committee of Lee Gil Ya Cancer and Diabetes Institute of Gachon University (#LCDI-2014-0020).

### 4.5. Animal Heart Tissue Preparation

Animals were sacrificed at sham point and 1 h, 3 h, 6 h, 12 h, 1 day, 3 days, 5 days, 7 days, 10 days, and 14 days after surgery and at 28 days following cell transplantation. Hearts were harvested and perfused through the right carotid artery with PBS and ice-cold 4% paraformaldehyde. Tissues were fixed in 4% paraformaldehyde (PFA, Sigma-Aldrich, 158127) at 4 °C overnight then transferred to dehydration procedure. After dehydration, tissues were cleared with xylene—2 times each for 1.5 h and embedded in paraffin at 60 °C. Paraffin-embedded heart tissues were sectioned at 7 µm thicknesses.

### 4.6. Evaluation of Infarct Size

Masson’s trichrome staining was performed to detect the infarction size, anterior wall thickness, and percent of fibrosis. Masson’s trichrome-stained sections were captured by light microscopy and the collagen-delegated infarction percentage was calculated and analyzed by a blinded investigator. The size of the infarct area and other parameters were measured on the middle horizontal sections between the point of ligation and the apex of the heart. The calculation formula used for the infarct size was the following [59]:


% infarct size = (infarct areas/total left ventricle (LV area)) × 100

### 4.7. Immunostaining

Tissues were deparaffinized in xylene for 10 min at room temperature and washed in PBS five times at the same time and incubated with the primary antibody (Table 1) specific to each target protein. After overnight incubation at 4 °C, the excess antibodies were washed with PBS, followed by incubation with a secondary fluorescent antibody (Table 1) at room temperature for 1 h. Nuclei were counterstained with DAPI (4′6-diamino-2-phenylindole; 1 μg/mL, Invitrogen, D1306) for 20 s at room temperature. After washing with PBS, cover slides were mounted using Vectashield mounting media (Vector Laboratories, H-1000; Newark, CA, USA) and images were analyzed using an LSM 710 confocal microscope at the same setting and same time (Carl Zeiss, Jena, Germany).

### 4.8. DAB (3,3′-Diaminobenzidine) Staining and Cell Counting

Tissues were deparaffinized in xylene at room temperature for 10 min, followed by dehydration in a graded ethanol series (100% ethanol, 3 min; 90% ethanol, 1 min; 80% ethanol, 1 min; 70% ethanol, 3 min; and water, 30 s). The heat-induced antigen retrieval step was performed in Tris-EDTA (Sigma-Aldrich, E9884; Saint Louis, MO, USA) buffer by boiling at 100 °C. Tissues were rinsed with cold distilled water and 0.1% Tween-PBS for 5 min and washed in 1X PBS five times. All tissue slides were immersed into 3% H_2_O_2_ (Sigma-Aldrich, 216763) for 20 min at room temperature to reduce endogenous peroxidase activity and blocked by 5% bovine serum albumin (BSA, MP Biomedicals, 160069; Solon, OH, USA) for 1 h at room temperature. Tissues were washed in 1X PBS three times and incubated with primary antibody (Iba-1, Abcam, ab5076) at 4 °C overnight. The tissues were washed again with PBS. ABC solution (Vector laboratories, PK6101; Newark, CA, USA) was added to tissues, and they were incubated at room temperature for 1 h. After washing with 1X PBS, the biotinylated secondary antibody was incubated for 1 h at room temperature. Tissues were washed again in PBS three times and incubated with DAB (Sigma-Aldrich, D5637; Saint Louis, MO, USA) solution at room temperature for 1 min. Finally, tissues were washed in a graded ethanol series (70%, 30 s; 80%, 30 s; 95% 30 s; 100% 30 s; and 100% Xylene 5 min). The stained slides were mounted with DPX mounting medium (Sigma-Aldrich, 06522; Saint Louis, MO, USA) for microscopic image analysis. Three replicates of the number of activated macrophages were counted under 200X magnification, and the average number was used for further analysis. 

### 4.9. The Terminal Deoxynucleotidyl Transferase dUTP Nick End Labeling (TUNEL) Assay

Paraffin-embedded heart tissues were deparaffinized in xylene for 10 min at room temperature, washed in 1X PBS five times, then incubated with permeabilization solution (0.1% Triton X-100 (Amresco, 0694; Solon, OH, USA), 0.1% Sodium citrate (Sigma-Aldrich, S1804, Saint Louis, MO, USA) for 2 min on ice. After permeabilization, tissues were rinsed 3 times with PBS. TUNEL reaction mixture (Roche, 12156792910; Mannheim, Germany) was added to tissues and incubated in a humidified atmosphere for 1 h at 37 °C in the dark. Tissues were washed again in PBS 3 times and coverslips were mounted onto glass slides using the Vectashield mounting medium (Vector Laboratories, H-1000; Newark, CA, USA). 

### 4.10. Co-Immunoprecipitation

Total cell lysates were prepared in lysis buffer (1 M Tris (pH 7.5, Amresco, 0497), 5 M NaCl (Sigma-Aldrich, S5886), 10% NP-40 (Fluka, 56741), 10% sodium deoxycholate (Sigma-Aldrich, 30970), and protease inhibitor cocktail (Roche, 05892970001)) followed by sonication. The lysates were centrifuged at 17,000× *g* for 20 min at 4 °C, and the supernatant was incubated overnight with the albumin antibody at 4 °C under constant head-to-tail rotation. Immunoprecipitants were collected with Protein G agarose beads (Invitrogen, 15918-014). The beads were washed in a lysis buffer (1 M Tris (pH 7.5, Amresco, 0497), 5 M NaCl (Sigma-Aldrich, S5886), 10% NP-40 (Sigma, 56741; Buchs, Switzerland), 10% sodium deoxycholate (Sigma-Aldrich, 30970; Saint Louis, MO, USA) and protease inhibitor cocktail (Roche, 05892970001; Mannheim, Germany)), and the immunoprecipitants were resuspended in a 1X SDS sample buffer. Equal amounts (10 µL) of protein were separated on 10% polyacrylamide gels and transferred to a nitrocellulose membrane (Millipore) at 200 mA for 2 h. Non-specific antibody binding was blocked by 5% non-fat skim milk at room temperature for 1 h. Membranes were incubated with primary protein-specific antibodies at 4 °C overnight and a secondary antibody at room temperature for 1 h. After washing several times, proteins were detected by enhanced chemiluminescence (ECL).

### 4.11. Enzyme-Linked Immunosorbent Assay (ELISA)

First, 96-well microplates were coated with 1 μg/mL of albumin antibody in 100 mM carbonate/bicarbonate (Sigma-Aldrich, S2127, S5761) buffer (pH 9.6) overnight at 4 °C. After washing the plates twice with PBS, the remaining protein-binding sites were blocked by adding 5% skim milk (Sigma, 70166; Buchs, Switzerland) at 4 °C overnight. Then, they were rinsed again with PBS and the samples of different group extracts were added to the plates and incubated for 90 min at 37 °C. Incubation at room temperature for 2 h was performed with 1 μg/mL of AGE antibody followed by rinsing with PBS. A horseradish peroxidase-conjugated secondary antibody was incubated with the samples after washing the plates with PBS. After adding the substrate 3,3′5,5′-tetramethylbenzidiine solution (Sigma-Aldrich, T0565) and incubating for 30 min, an equal volume of stop solution of 2 N H_2_SO_4_ (Sigma-Aldrich, 7664-93-9; Saint Louis, MO, USA) was added, and optical density at 450 nm was determined. 

### 4.12. Densitometry and Statistical Analysis

The densitometry intensity of each immunoreactivity band was determined using Image-Pro gel digitizing software. All data shown in this study represent results from at least three independent experiments. Statistical analyses were performed using the *t*-test or ANOVA followed by Tukey’s post hoc test, and *p* < 0.05 was considered significant (*, *p* < 0.05; **, *p* < 0.001; ***, *p* < 0.0001).

## 5. Conclusions

The present study demonstrated that recruited macrophages are capable of secreting AGE-albumin under AMI conditions, and consequently that AGE-RAGE-dependent cellular signaling promotes the apoptosis of cardiomyocytes during the pathogenesis of AMI. Based on the toxicity of the AGE-RAGE system in cardiovascular disorders, AGE-RAGE-targeted therapeutic approaches are extensively studied, and the application of sRAGE is considered as one of the most suitable targets due to that it possesses natural features of the RAGE antagonist and due to promising results in AGE-RAGE-induced disorders. Our results demonstrate that the application of MSCs as unremitted carriers of sRAGE during post-AMI incidence could bring increased success in clinical management and treatment of patients suffering from cardiovascular disorders. However, it is necessary to perform further research to demonstrate a safe and effective dosage of sRAGE, as well as improve issues such as cell homing and HLA match.

## Figures and Tables

**Table 1:** List of antibodies used in this study.

Antigen	Host	Company (Cat No)	Working Dilution
Albumin	Mouse	Abcam (ab10241)	IF-1:100, WB-1:1000
AGE	Rabbit	Abcam (ab23722)	IF-1:200, WB-1:3000
Iba-1	Goat	Abcam (ab5076)	IF-1:100
RAGE	Goat	Abcam (ab7764)	IF-1:400, WB-1:4000
FLAG	Rabbit	Sigma-aldrich (F7425)	WB-1:1000
p38	Rabbit	Cell signaling (9212L)	WB-1:1000
pp38	Rabbit	Cell signaling (9211S)	WB-1:1000
ERK1/2	Rabbit	Cell signaling (9102S)	WB-1:1000
pERK1/2	Rabbit	Cell signaling (4377S)	WB-1:1000
SAPK/JNK	Rabbit	Cell signaling (9252S)	WB-1:1000
pSAPK/JNK	Rabbit	Cell signaling (9251S)	WB-1:1000
Β-Actin	Rabbit	Abcam (ab8227)	WB-1:1000
Peroxidase-labeled anti-mouse IgG	Mouse	Vector (PI2000)	WB-1:5000
Peroxidase labeled anti-rabbit IgG	Rabbit	Vector (PI 1000)	WB-1:5000
Peroxidase-labeled anti-goat IgG	Goat	Vector (PI9500)	WB-1:5000
Alexa Fluor 555 donkey anti-rabbit IgG	Rabbit	Invitrogen (A31572)	IF-1:500
Alexa Fluor 633 goat anti-rabbit IgG	Rabbit	Invitrogen (A21070)	IF-1:500
Alexa Fluor 555 donkey anti-goat IgG	Goat	Invitrogen (A21432)	IF-1:500
Alexa Fluor 488 donkey anti mouse	Mouse	Invitrogen (A11001)	IF-1:500

IF—immunofluorescence, WB—Western blot.

## References

[1] Bayarsaikhan D., Bayarsaikhan G., Lee B. (2021). Recent advances in stem cells and gene editing: Drug discovery and therapeutics. Prog. Mol. Biol. Transl. Sci..

[2] Mc Namara K., Alzubaidi H., Jackson J.K. (2019). Cardiovascular disease as a leading cause of death: How are pharmacists getting involved?. Integr. Pharm. Res. Pract..

[3] Buja L.M. (2005). Myocardial ischemia and reperfusion injury. Cardiovasc Pathol..

[4] Frank A., Bonney M., Bonney S., Weitzel L., Koeppen M., Eckle T. (2012). Myocardial ischemia reperfusion injury: From basic science to clinical bedside. Semin. Cardiothorac. Vasc. Anesth..

[5] Hausenloy D.J., Yellon D.M. (2013). Myocardial ischemia-reperfusion injury: A neglected therapeutic target. J. Clin. Investig..

[6] Li Z., Dong X., Tian M., Liu C., Wang K., Li L., Liu Z., Liu J. (2020). Stem cell-based therapies for ischemic stroke: A systematic review and meta-analysis of clinical trials. Stem. Cell Res. Ther..

[7] Goradel N.H., Hour F.G., Negahdari B., Malekshahi Z.V., Hashemzehi M., Masoudifar A., Mirzaei H. (2018). Stem Cell Therapy: A New Therapeutic Option for Cardiovascular Diseases. J. Cell Biochem..

[8] Wernly B., Mirna M., Rezar R., Prodinger C., Jung C., Podesser B.K., Kiss A., Hoppe U.C., Lichtenauer M. (2019). Regenerative Cardiovascular Therapies: Stem Cells and Beyond. Int. J. Mol. Sci..

[9] Müller P., Lemcke H., David R. (2018). Stem Cell Therapy in Heart Diseases—Cell Types, Mechanisms and Improvement Strategies. Cell. Physiol. Biochem..

[10] Nguyen P.K., Rhee J.W., Wu J.C. (2016). Adult Stem Cell Therapy and Heart Failure, 2000 to 2016: A Systematic Review. JAMA Cardiol..

[11] Kawaguchi M., Takahashi M., Hata T., Kashima Y., Usui F., Morimoto H., Izawa A., Takahashi Y., Masumoto J., Koyama J. (2011). Inflammasome activation of cardiac fibroblasts is essential for myocardial ischemia/reperfusion injury. Circulation.

[12] Weinberger T., Schulz C. (2015). Myocardial infarction: A critical role of macrophages in cardiac remodeling. Front. Physiol..

[13] Frangogiannis N.G., Smith C.W., Entman M.L. (2002). The inflammatory response in myocardial infarction. Cardiovasc. Res..

[14] Niu J., Kolattukudy P.E. (2009). Role of MCP-1 in cardiovascular disease: Molecular mechanisms and clinical implications. Clin. Sci..

[15] Morimoto H., Takahashi M., Izawa A., Ise H., Hongo M., Kolattukudy P.E., Ikeda U. (2006). Cardiac overexpression of monocyte chemoattractant protein-1 in transgenic mice prevents cardiac dysfunction and remodeling after myocardial infarction. Circ. Res..

[16] Kakio T., Matsumori A., Ono K., Ito H., Matsushima K., Sasayama S. (2000). Roles and relationship of macrophages and monocyte chemotactic and activating factor/monocyte chemoattractant protein-1 in the ischemic and reperfused rat heart. Lab. Investig..

[17] Son M., Kang W.C., Oh S., Bayarsaikhan D., Ahn H., Lee J., Park H., Lee S., Choi J., Lee H.S. (2017). Advanced glycation end-product (AGE)-albumin from activated macrophage is critical in human mesenchymal stem cells survival and post-ischemic reperfusion injury. Sci. Rep..

[18] Bayarsaikhan G., Bayarsaikhan D., Lee J., Lee B. (2022). Targeting Scavenger Receptors in Inflammatory Disorders and Oxidative Stress. Antioxidants.

[19] Byun K., Bayarsaikhan E., Kim D., Kim C.Y., Mook-Jung I., Paek S.H., Kim S.U., Yamamoto T., Won M.H., Song B.J. (2012). Induction of neuronal death by microglial AGE-albumin: Implications for Alzheimer’s disease. PLoS ONE.

[20] Rungratanawanich W., Qu Y., Wang X., Essa M.M., Song B.J. (2021). Advanced glycation end products (AGEs) and other adducts in aging-related diseases and alcohol-mediated tissue injury. Exp. Mol. Med..

[21] Bayarsaikhan G., Bayarsaikhan D., Oh P.C., Kang W.C., Lee B. (2021). CUPRAC-Reactive Advanced Glycation End Products as Prognostic Markers of Human Acute Myocardial Infarction. Antioxidants.

[22] Byun K., Yoo Y., Son M., Lee J., Jeong G.B., Park Y.M., Salekdeh G.H., Lee B. (2017). Advanced glycation end-products produced systemically and by macrophages: A common contributor to inflammation and degenerative diseases. Pharmacol. Ther..

[23] Neviere R., Yu Y., Wang L., Tessier F., Boulanger E. (2016). Implication of advanced glycation end products (Ages) and their receptor (Rage) on myocardial contractile and mitochondrial functions. Glycoconj. J..

[24] Yu L., Zhao Y., Xu S., Ding F., Jin C., Fu G., Weng S. (2013). Advanced Glycation End Product (AGE)-AGE Receptor (RAGE) System Upregulated Connexin43 Expression in Rat Cardiomyocytes via PKC and Erk MAPK Pathways. Int. J. Mol. Sci..

[25] Cho H.J., Xie C., Cai H. (2018). AGE-induced neuronal cell death is enhanced in G2019S LRRK2 mutation with increased RAGE expression. Transl. Neurodegener..

[26] Aleshin A., Ananthakrishnan R., Li Q., Rosario R., Lu Y., Qu W., Song F., Bakr S., Szabolcs M., D’Agati V. (2008). RAGE modulates myocardial injury consequent to LAD infarction via impact on JNK and STAT signaling in a murine model. Am. J. Physiol. Heart Circ. Physiol..

[27] Yan S.F., Ramasamy R., Schmidt A.M. (2010). The RAGE axis: A fundamental mechanism signaling danger to the vulnerable vasculature. Circ. Res..

[28] Xu Y., Toure F., Qu W., Lin L., Song F., Shen X., Rosario R., Garcia J., Schmidt A.M., Yan S.F. (2010). Advanced glycation end product (AGE)-receptor for AGE (RAGE) signaling and up-regulation of Egr-1 in hypoxic macrophages. J. Biol. Chem..

[29] Shang L., Ananthakrishnan R., Li Q., Quadri N., Abdillahi M., Zhu Z., Qu W., Rosario R., Touré F., Yan S.F. (2010). RAGE modulates hypoxia/reoxygenation injury in adult murine cardiomyocytes via JNK and GSK-3beta signaling pathways. PLoS ONE.

[30] Yue J., López J.M. (2020). Understanding MAPK Signaling Pathways in Apoptosis. Int. J. Mol. Sci..

[31] Chen X., Zhang X., Kubo H., Harris D.M., Mills G.D., Moyer J., Berretta R., Potts S.T., Marsh J.D., Houser S.R. (2005). Ca^2+^ influx-induced sarcoplasmic reticulum Ca^2+^ overload causes mitochondrial-dependent apoptosis in ventricular myocytes. Circ. Res..

[32] Liu Z., Xu Q., Yang Q., Cao J., Wu C., Peng H., Zhang X., Chen J., Cheng G., Wu Y. (2019). Vascular peroxidase 1 is a novel regulator of cardiac fibrosis after myocardial infarction. Redox Biol..

[33] Byun K., Bayarsaikhan E., Kim D., Son M., Hong J., Jeong G.B., Paek S.H., Won M.H., Lee B. (2012). Activated microglial cells synthesize and secrete AGE-albumin. Anat. Cell Biol..

[34] Byun K., Bayarsaikhan D., Bayarsaikhan E., Son M., Oh S., Lee J., Son H.I., Won M.H., Kim S.U., Song B.J. (2014). Microglial AGE-albumin is critical in promoting alcohol-induced neurodegeneration in rats and humans. PLoS ONE.

[35] Bayarsaikhan E., Bayarsaikhan D., Lee J., Son M., Oh S., Moon J., Park H.J., Roshini A., Kim S.U., Song B.J. (2016). Microglial AGE-albumin is critical for neuronal death in Parkinson’s disease: A possible implication for theranostics. Int. J. Nanomed..

[36] Frangogiannis N.G. (2019). Cardiac fibrosis: Cell biological mechanisms, molecular pathways and therapeutic opportunities. Mol. Aspects Med..

[37] Hu S., Yang M., Huang S., Zhong S., Zhang Q., Ding H., Xiong X., Hu Z., Yang Y. (2022). Different Roles of Resident and Non-resident Macrophages in Cardiac Fibrosis. Front. Cardiovasc. Med..

[38] Yashima H., Terasaki M., Sotokawauchi A., Matsui T., Mori Y., Saito T., Osaka N., Kushima H., Hiromura M., Ohara M. (2020). AGE-RAGE Axis Stimulates Oxidized LDL Uptake into Macrophages through Cyclin-Dependent Kinase 5-CD36 Pathway via Oxidative Stress Generation. Int. J. Mol. Sci..

[39] Zhang L., He J., Wang J., Liu J., Chen Z., Deng B., Wei L., Wu H., Liang B., Li H. (2021). Knockout RAGE alleviates cardiac fibrosis through repressing endothelial-to-mesenchymal transition (EndMT) mediated by autophagy. Cell Death Dis..

[40] Wang Q., Zhu G., Cao X., Dong J., Song F., Niu Y. (2017). Blocking AGE-RAGE Signaling Improved Functional Disorders of Macrophages in Diabetic Wound. J. Diabetes Res..

[41] Senatus L.M., Schmidt A.M. (2017). The AGE-RAGE Axis: Implications for Age-Associated Arterial Diseases. Front. Genet..

[42] Zhang F., Su X., Huang G., Xin X.F., Cao E.H., Shi Y., Song Y. (2017). sRAGE alleviates neutrophilic asthma by blocking HMGB1/RAGE signalling in airway dendritic cells. Sci. Rep..

[43] Jensen L.J., Lindberg S., Hoffmann S., Iversen A.Z., Pedersen S.H., Møgelvang R., Galatius S., Flyvbjerg A., Jensen J.S., Bjerre M. (2015). Dynamic changes in sRAGE levels and relationship with cardiac function in STEMI patients. Clin. Biochem..

[44] Selejan S.R., Hewera L., Hohl M., Kazakov A., Ewen S., Kindermann I., Böhm M., Link A. (2017). Suppressed MMP-9 Activity in Myocardial Infarction-Related Cardiogenic Shock Implies Diminished Rage Degradation. Shock.

[45] Grauen Larsen H., Marinkovic G., Nilsson P.M., Nilsson J., Engström G., Melander O., Orho-Melander M., Schiopu A. (2019). High Plasma sRAGE (Soluble Receptor for Advanced Glycation End Products) Is Associated with Slower Carotid Intima-Media Thickness Progression and Lower Risk for First-Time Coronary Events and Mortality. Arterioscler. Thromb. Vasc. Biol..

[46] Liu Y., Guo X., Zhang J., Han X., Wang H., Du F., Zeng X., Guo C. (2021). Protective Effects of the Soluble Receptor for Advanced Glycation End-Products on Pyroptosis during Myocardial Ischemia-Reperfusion. Oxid. Med. Cell Longev..

[47] Zhang X., Xie J., Sun H., Wei Q., Nong G. (2022). sRAGE Inhibits the Mucus Hypersecretion in a Mouse Model with Neutrophilic Asthma. Immunol. Investig..

[48] Prasad K. (2020). AGE-RAGE Stress in the Pathophysiology of Atrial Fibrillation and Its Treatment. Int. J. Angiol..

[49] Quade-Lyssy P., Kanarek A.M., Baiersdörfer M., Postina R., Kojro E. (2013). Statins stimulate the production of a soluble form of the receptor for advanced glycation end products. J. Lipid. Res..

[50] Falcone C., Bozzini S., D’Angelo A., Matrone B., Colonna A., Benzi A., Paganini E.M., Falcone R., Pelissero G. (2013). Plasma levels of soluble receptor for advanced glycation end products and coronary atherosclerosis: Possible correlation with clinical presentation. Dis. Markers.

[51] Lee J., Bayarsaikhan D., Arivazhagan R., Park H., Lim B., Gwak P., Jeong G.B., Lee J., Byun K., Lee B. (2019). CRISPR/Cas9 Edited sRAGE-MSCs Protect Neuronal Death in Parkinson’s Disease Model. Int. J. Stem Cells.

[52] Park M.J., Lee S.H., Moon S.J., Lee J.A., Lee E.J., Kim E.K., Park J.S., Lee J., Min J.K., Kim S.J. (2016). Overexpression of soluble RAGE in mesenchymal stem cells enhances their immunoregulatory potential for cellular therapy in autoimmune arthritis. Sci. Rep..

[53] Attar A., Monabati A., Montaseri M., Vosough M., Hosseini S.A., Kojouri J., Abdi-Ardekani A., Izadpanah P., Azarpira N., Pouladfar G. (2022). Transplantation of mesenchymal stem cells for prevention of acute myocardial infarction induced heart failure: Study protocol of a phase III randomized clinical trial (Prevent-TAHA8). Trials.

[54] Attar A., Bahmanzadegan Jahromi F., Kavousi S., Monabati A., Kazemi A. (2021). Mesenchymal stem cell transplantation after acute myocardial infarction: A meta-analysis of clinical trials. Stem. Cell Res. Ther..

[55] van den Akker F., Deddens J.C., Doevendans P.A., Sluijter J.P. (2013). Cardiac stem cell therapy to modulate inflammation upon myocardial infarction. Biochim. Biophys. Acta.

[56] Suzuki E., Fujita D., Takahashi M., Oba S., Nishimatsu H. (2017). Therapeutic Effects of Mesenchymal Stem Cell-Derived Exosomes in Cardiovascular Disease. Adv. Exp. Med. Biol..

[57] Milutinovic P.S., Englert J.M., Crum L.T., Mason N.S., Ramsgaard L., Enghild J.J., Sparvero L.J., Lotze M.T., Oury T.D. (2014). Clearance kinetics and matrix binding partners of the receptor for advanced glycation end products. PLoS ONE.

[58] Suchal K., Malik S., Khan S.I., Malhotra R.K., Goyal S.N., Bhatia J., Kumari S., Ojha S., Arya D.S. (2017). Protective effect of mangiferin on myocardial ischemia-reperfusion injury in streptozotocin-induced diabetic rats: Role of AGE-RAGE/MAPK pathways. Sci. Rep..

[59] Takagawa J., Zhang Y., Wong M.L., Sievers R.E., Kapasi N.K., Wang Y., Yeghiazarians Y., Lee R.J., Grossman W., Springer M.L. (2007). Myocardial infarct size measurement in the mouse chronic infarction model: Comparison of area- and length-based approaches. J. Appl. Physiol..

